# Surgical treatment for achilles tendinopathy – a systematic review

**DOI:** 10.1186/s12891-016-1061-4

**Published:** 2016-05-10

**Authors:** Heinz Lohrer, Sina David, Tanja Nauck

**Affiliations:** ESN – European Sportscare Network, Zentrum für Sportorthopädie, Borsigstrasse 2, 65205 Wiesbaden-Nordenstadt, Germany; Department of Sport and Sport Science, University of Freiburg, Schwarzwaldstraße 175, 79117 Freiburg, Germany; Deutsche Sporthochschule Köln, Am Sportpark Müngersdorf 6, 50933 Köln, Germany

**Keywords:** Systematic review, Achilles tendinopathy, Noninsertional, Midportion, Operative treatment

## Abstract

**Background:**

The purpose of this systematic review is to analyse the results of operative treatment for midportion Achilles tendinopathy and to provide evidence based recommendation for the indication of the individual published techniques.

**Methods:**

MEDLINE, Cochrane Database, ISI Web of Knowledge and Google databases (1945 till September 2014) were electronically searched. The quality of the included articles was evaluated using the Coleman Methodology Score. Success rates, patient satisfaction, and the complication rates were determined.

**Results:**

Twenty studies met our inclusion criteria. A total of 801 tendons were treated in 714 patients with open or minimally invasive techniques. The mean success rate was 83.4 %. Complications were reported in 6.3 % of the cases. The articles on minimally invasive techniques and open procedures reported on an average success rate of 83.6 % and 78.9 (*p* = 0.987). Patient satisfaction rates for minimally invasive techniques and open procedures were 78.5 % and 78.1 % (*p* = 0.211). The complication rate was 5.3 % for the minimally invasive techniques and 10.5 % for the open procedures (*p* = 0.053).

**Conclusion:**

We conclude that success rates of minimally invasive and open treatments are not different and that there is no difference in patient satisfaction but there is a tendency for more complications to occur in open procedures.

## Background

Midportion Achilles tendinopathy is a wide-spread disorder with a prevalence of 2.01 per 1,000 patients [1]. Its aetiology is thought to be associated with multiple factors including overuse, poor vascularity, a lack of flexibility, genetic makeup, gender, endocrine, a high body mass index or metabolic factors [1–7]. Historically, the terminology for midportion Achilles tendinopathy was not consistent. We adopted the recently suggested definition: “a clinical syndrome characterized by a combination of pain, swelling and impaired performance” [8]. Midportion Achilles tendinopathy is located about 2–6 cm proximal to the Achilles tendon insertion onto the calcaneus [6, 9]. The painful region coincides with the tendon area possessing the poorest blood supply [4, 5, 10].

The “tendon pathology continuum model” describes a discrepancy between load in relation to intrinsic factors like genetics, adiposity, cholesterol, and diabetes finally leading to degeneration and insufficient regenerative capability of an individual Achilles tendon [11]. In the literature, several hypotheses have been established to explain the cause of pain in Achilles tendinopathy [12]. Besides intratendineous degeneration (tendinosis), neovascularization and neurogenic inflammatory processes seem to play a major role with pain representing the “tip of the iceberg” [12].

Much has been published about the conservative treatment of midportion Achilles tendinopathy [13–16]. Conservative modalities include load modification, eccentric exercises, orthoses, massage, electrotherapy, cryotherapy, nonsteroidal anti-inflammatory drugs, extracorporeal shockwave therapy (ESWT), high volume and sclerosing injections [5, 17]. However, about 25 % of the patients present with remaining symptoms after conservative treatment [6, 7, 14–16, 18]. For these patients, operative intervention is indicated [5, 6, 17, 19]. According to the “tendon pathology continuum model” [11] and to recent pathogenic considerations [12] two principally different operative approaches and their combinations can be identified. Intratendineous lesions (tendinosis) or/and the pain producing or pain transmitting neurogenic structures outside the Achilles tendon are addressed. Specifically, the procedures address (a) removal of the abnormal tissue inside the Achilles tendon and the paratenon, (b) activation of the regenerative process by scarification of the Achilles tendon, (c) vascular disruption, (d) gastrocnemius recession to reduce the tension and therefore the overload of the Achilles tendon, and (e) if the quality of the tendinopathic Achilles tendon is poor (more severe cases), a transfer of an intact tendon (Flexor hallucis longus) can be performed [10, 20]. In 2001, a “critical review” for the operative treatment of midportion Achilles tendinopathy and in 2015 a systematic review for “outcomes for insertional and non-insertional Achilles tendinopathy surgery” were published [20, 21]. However, there is no systematic review available for operative treatment of midportion Achilles tendinopathy considering the different operative techniques.

The aim of this study is therefore, to systematically review the literature for operative treatment of midportion Achilles tendinopathy. It is hypothesized that operative approaches are effective for midportion Achilles tendinopathy not responding to first line conservative treatment. Additionally, it is questioned if a specific open or minimally invasive operative technique can be considered to be superior when comparing results and complications.

## Methods

### Search strategy

MEDLINE, Cochrane Database, ISI Web of Knowledge and Google were systematically searched by two reviewers using the terms: (achilles tendinopathy OR achilles tendopathy OR achillodynia OR achillodynie OR tendinopathy OR tendo achilles OR achilles tendon OR achilles tendinosis OR achilles tendonosis OR tendinosis) AND ((midportion OR mid-portion OR non-insertional OR main body OR central core OR noninsertional OR mid portion OR percutaneous longitudinal tenotomy OR flexor hallucis longus transfer OR FHL OR gastrocnemius recession OR gastrocnemius lengthening OR paratenon release OR achilles tendinoscopy OR debridement) AND (surgery OR surgical OR surg OR operative OR operation OR treatment) NOT calcaneal bursitis OR bursitis OR hagland OR haglund OR insertional)) Filters: Publication date to 2014/09/31. The Cochrane Database of Clinical and Randomized Controlled Trials was additionally searched using the term “Achilles”. The Google Scholar search was performed by using the keywords “Achilles tendinopathy” surgery -“calcaneal bursitis” -bursitis -hagland -haglund –insertional.

### Data collection and study selection

The procedure was based on the PRISMA guidelines for reporting systematic reviews and meta‐analyses [22]. Two reviewers independently evaluated the titles and abstracts of the identified publications and the selected full text manuscripts in an unblinded standardized manner and excluded irrelevant articles (reviews, cadaver studies, technical descriptions). Disagreements between reviewers were resolved by consensus. Articles in English and German language were included. Differences remaining between the reviewers concerning inclusion of studies were discussed and consensus was obtained (3 cases).

### Inclusion criteria

We included prospective clinical studies reporting on the subjective, clinical, or functional outcome of operative treatment for midportion Achilles tendinopathy. Studies had to characterize the clinical syndrome by pain, swelling and impaired performance. Results from studies reporting on adult patients (age over 18 years) with midportion Achilles tendinopathy were included.

### Exclusion criteria

Studies, dealing with the treatment of Achilles tendon ruptures, insertional Achilles tendinopathy, retrocalcaneal bursitis/Haglund’s disease and superficial calcaneal bursitis were excluded. Studies which used an inconsistent terminology were excluded since the treated condition was not unequivocally midportion Achilles tendinopathy. We didn’t consider case series with less than five patients. Articles on expert opinion, reviews, those with retrospective design and unidentified outcome measures were also excluded. If different techniques were reported in one study and the results were not specified with respect to the used technique, we decided to exclude these data for the subgroup analysis. Studies which did not report on one of the required main outcome measures were not included in the specific calculations.

### Data extraction and quality assessment

We extracted outcome data using the Coleman methodology scale (CMS) [23]. By this it was possible to measure the methodological quality of the included studies. The score ranges between 0 and 100. A score of 100 represents a perfect study design that largely avoids the influence of chance, different biases, and confounding factors. As primary outcome criteria the results of the studies were classified referring to the “functional classification of postsurgical outcome for Achilles tendinopathy” as excellent, good, fair, or poor [20, 24]. This categorical and disease specific rating scale was developed for evaluation of outcome after Achilles tendinopathy operations. An excellent result indicates no residual symptoms and unlimited sport performance. Good means full sport ability but “some stiffness after strenuous activities”. Fair is rated when there is improvement but still “stiffness and aching” related to sport activities. “No improvement at all” is considered poor. Even if the reliability and validity of this tool has not formally been analysed, it has been used to evaluate outcome in most of our reviewed studies. Excellent and good results sum up to the success rate [20]. Previous Achilles tendinopathy reviews reported improvement of methodological quality and a negative correlation between success rates during the reviewed periods [20, 21]. Respectively, we also correlated the CMS with operative success rate (%) and the year of publication. Secondary outcome criteria were patient’s satisfaction and complications related to the performed operations. Patient’s satisfaction is a subjective rating of the patients. The complication rate sums up wound infections, scar hypersensitivity in the operative field, hypertrophic scars, skin necrosis and fibrotic reactions, the need of further surgery, or Achilles tendon ruptures, as well as deep vein thrombosis and/or lung embolism. For analysis the results of the included studies were pooled referring to the used techniques (see grouping). This led to a main group analysis (open vs. minimally invasive) and to a subgroup analysis (open release of adhesions with or without resection of the paratenon, open debridement of tendinopathic areas through a central longitudinal tenotomy, flexor hallucis longus (FHL) transfer, longitudinal tenotomy, gastrocnemius lengthening or recession, percutaneous longitudinal tenotomy, minimally invasive debridement).

### Grouping

In the initial part of the analyses and based on a recently published instructional review [15] we pooled the extracted data in two groups (open techniques and minimally invasive techniques). A technique was classified as minimally invasive, if the length of a single incision was less than 1 inch (2.5 cm). The open procedures group included longitudinal tenotomies with debridement of the diseased area of the tendon with or without tendon augmentation, and gastrocnemius lengthening or recession. The minimally invasive group included percutaneous longitudinal tenotomies, endoscopic debridement or scraping with or without the augmentation of the flexor hallucis longus or the plantaris tendon, and minimally invasive gastrocnemius lengthening or recession.

For the subgroup analysis we pooled the data in six groups: open peritendineous debridement = open release of adhesion with or without resection of the paratenon, open intratendineous debridement = open debridement of tendinopathic areas through a central longitudinal tenotomy, FHL transfer/augmentation, gastrocnemius lengthening or recession, percutaneous longitudinal tenotomy, and minimally invasive paratenon debridement.

### Statistical analysis

For the statistical analysis IBM SPSS 21 was used. Operative methods were compared by descriptive statistics (weighted means and standard deviations). All data were checked for normal distribution. The level of significance was defined at *p* = 0.05. We compared the results of the different techniques in two steps. In a first step the results of open and minimally invasive techniques (main group/outcome) were compared. In a second step subgroups (six specific techniques) were compared (open peritendineous debridement, open intratendineous debridement, FHL tendon transfer/augmentation, gastrocnemius lengthening or recession, percutaneous longitudinal tenotomy, and minimally invasive paratenon debridement). A Breslow-Day-Test was used to compare the odds’ ratio of the correlation between the different techniques. After that a Mantel-Haenszel-chi-squared test was used to compare the outcome of the different techniques. A Bonferroni-Holm-Test was used for the post-hoc test. Pearson correlation was calculated between the CMS and the success rate and the CMS and the year of publication.

## Results

### Study selection

A total of 4453 studies were electronically identified for inclusion in the review. After adjusting for duplicates 4378 potentially relevant studies remained. After reviewing the titles and abstracts 4302 of these studies were discarded because it appeared that these papers clearly did not meet the criteria. The full text of the remaining 76 papers was obtained and examined in more detail. Fifty-six studies did not meet the inclusion criteria as described. The hand search of the references identified one more relevant article which was not detected from the electronic search. Twenty-one articles were considered to be relevant and the respective full texts were further analysed. Three authors of the included studies were contacted by email to obtain further detailed information which was not presented in their publications [18, 25, 26].

**Fig. 1:**
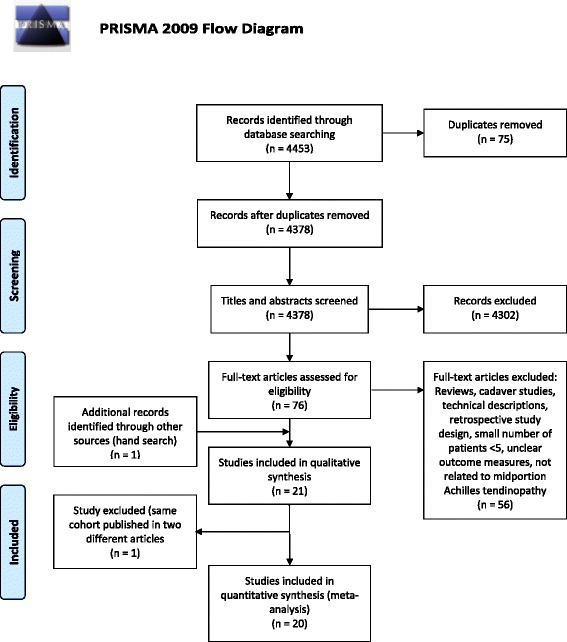
Prisma flowchart [22] of the data collection and study selection progress

One group of researchers [27] published results once in 1997 and an update in 2013 [28]. We only included the updated results. Finally, 20 articles remained and served as the database for this review (Fig. 1) [4, 18, 19, 24–26, 28–41].

### Population characteristics

The total number of patients with Achilles tendinopathy in the 20 included studies was 714 and 801 tendons were treated. Eight of the included studies treated 388 patients with bilateral midportion Achilles tendinopathy [4, 24, 25, 30, 32, 36, 38, 41]. The mean age of the patients was 46.6 years (range 28.7 to 61). There were 61 % males and 39 % females. Seven studies did not report patient characteristics or did not state them clearly regarding age and gender [18, 26, 28, 31, 32, 34]. Three studies reported different results for variable populations [25, 34, 35]. One compared the results of female and male patients and reported both a higher success rate and a lower complication rate in males [35]. In two other studies the authors showed that athletic patients recovered with a higher success rate accompanied by a lower complication rate compared to non-athletic patients [25, 34].

### Preoperative treatment

Preoperatively, all patients underwent conservative treatment for at least three months. The reported conservative treatments included immobilization, eccentric exercise, stretching, cryotherapy, ultrasound therapy, laser therapy, orthotics, extracorporeal shock wave therapy, sclerosing injections, and anti-inflammatory medication [4, 18, 19, 24–26, 28–41].

### Grouping

The open procedure group (542 patients) included longitudinal tenotomies with debridement of the diseased area of the tendon with or without tendon augmentation (537 patients) [4, 18, 19, 24, 29, 32, 34, 35, 37, 41] and gastrocnemius lengthening or recession (5 patients, Table 1) [31]. The minimally invasive group (172 patients) included percutaneous longitudinal tenotomies (47 patients) [28, 40], endoscopic debridement or scraping with or without augmentation of the flexor hallucis longus or the plantaris tendon (111 patients) [25, 26, 33, 38, 39, 42] and minimally invasive gastrocnemius lengthening or recession (14 patients) [30].

**Table 1:** Results of the reviewed literature for open and minimally invasive procedures

	Year	CMS	N	Age	FU	SR	PS	CR
Open techniques								
Rolf et al. [4]	1997	60	58	40	25	75	n.s	13
Nelen et al. [24]	1989	66	91	30	48	85	n.s	7
Cottom et al. [29]	2008	81	62	61	27	n.s	95	23
Wilcox et al. [41]	2000	58	17	61	14	75	n.s	0
Martin et al. [37]	2005	84	44	58	41	91	86	11
Alfredson et al. [19]	2007	53	10	45	6	100	80	10
Lohrer & Nauck [32]	2014	81	34	50	12	97	n.s	8
Paavola et al. [18]	2002	59	42	42	7	83	87	6
Maffulli et al. [34]	2006	75	93	n.s	37	66	68	9
Maffulli et al. [35]	2008	75	86	n.s	40	73	69	10
Gurdezi et al. [31]	2013	70	5	45	30	n.s	66	n.s
		69.3 ± 10.7	542	45.6 ± 10.5	32.0 ± 14.6	78.9 ± 11.5	78.1 ± 11.3	10.5 ± 5.9
Minimally invasive techniques								
Duthon et al. [30]	2011	64	14	42	24	79	60	0
Maquirriain [42]	2013	79	24	46	92	100	n.s	7
Lui [33]	2012	69	5	46	20	80	n.s	0
Thermann et al. [39]	2009	69	8	52	6	80	80	0
Maffulli et al. [28]	2013	84	39	45	204	77	77	18
Pearce et al. [26]	2011	62	11	37	30	n.s	72	0
Vega et al. [40]	2008	69	8	43	27	100	n.s	0
Alfredson [25]	2011	73	16	47	18	74	74	3
Ruergård & Alfredson [38]	2014	79	47	52	12	n.s	88	0
		72.0 ± 7.4	172	46.7 ± 4.7	70.0 ± 63.6	83.6 ± 10.9	78.5 ± 9.3	5.3 ± 6.1

Year of publication (Year), Coleman Methodological Score (CMS), Number of patients (N), Age (years), Followup period (FU) in months , Success rate (SR) in %, Patient satisfaction (PS) in %, and Complication rates (CR) in %. Values for FU, SR, and PS of the individual studies are presented as means. N.s. = not specified. Summarized information of the open and minimally invasive techniques studies are presented in the last lines of the respective sections (CMS = unweighted mean. Age, FU, SR, PS, and CR = weighted means ± SD)

The subgroups analyses revealed 109 patients treated with open peritendineous debridement [18, 24], 96 patients had open intratendineous debridement [18, 19, 24, 32], 152 patients had FHL transfer/augmentation [24, 29, 33, 37, 41], 19 patients had gastrocnemius lengthening or recession [30, 31], 126 patients had percutaneous longitudinal tenotomy [25, 28], and 106 patients had minimally invasive paratenon debridement [25, 26, 38, 40, 42] (Table 2). Two studies treated patients with different techniques but did not clearly enough state the results and were therefore not included in the subgroup analysis [34, 35]. Two studies presented cohorts treated with different operative techniques (percutaneous longitudinal tenotomy and minimally invasive paratenon debridement) [24, 25]. One of these studies specified the number of patients only for the whole group. For the performed operative techniques only the numbers of Achilles tendons are presented. For this specific case, we decided to incorporate the number of treated Achilles tendons in our subgroup analyses [24].

**Table 2:** Results of the reviewed literature for the specific operative techniques

	Year	CMS	N	Age	FU	SR	PS	CR
Open peritendineous debridement								
Nelen et al. [24]^a^	1989	66	93	n.s.	n.s.	88	n.s.	n.s.
Paavola et al. [18]	2002	59	16	37	7	100	94	6
		62.5 ± 4.9	109	37	7	89.8 ± 8.5	94	6
Open intratendineous debridement								
Nelen et al. [24]^a^	1989	66	26	n.s.	n.s.	73	n.s.	n.s.
Alfredson et al. [19]	2007	53	10	45	6	100	80	10
Lohrer & Nauck [32]	2014	81	34	50	12	97	n.s.	8
Paavola et al. [18]	2002	59	26	46	7	73	79	27
		64.8 ± 12.1	96	47.8 ± 2.6	6.8 ± 4.9	84.3 ± 14.8	79.3 ± 0.7	15.3 ± 10.4
FHL transfer/augmentation								
Nelen et al. [24]	1989	66	24	n.s.	n.s.	87	n.s.	n.s.
Cottom et al. [29]	2008	81	62	61	27	n.s.	95	23
Wilcox et al. [41]	2000	58	17	61	14	75	n.s.	0
Martin et al. [37]	2005	84	44	58	41	91	86	11
Lui et al. [33]	2012	69	5	46	20	80	n.s.	0
		71.6 ± 10.8	152	59.4 ± 7.1	29.8 ± 11.6	86.3 ± 7.1	91.3 ± 6.4	14.9 ± 11.0
Gastrocnemius recession								
Duthon et al. [30]	2011	64	14	42	24	79	60	0
Gurdezi et al. [31]	2013	70	5	45	30	n.s.	66	n.s.
		67.0 ± 4.2	19	42.8 ± 2.1	25.6 ± 4.2	79.0	61.6 ± 4.2	0.0
Percutaneous longitudinal tenotomy								
Alfredson et al. [25]	2011	73	87	46	18	83	83	3
Maffulli et al. [28]	2013	84	39	45	204	77	77	18
		78.5 ± 7.8	126	45.7 ± 0.7	75.6 ± 131.6	81.1 ± 4.2	81.1 ± 4.2	7.6 ± 10.6
Minimally invasive paratenon debridement								
Maquirriain et al. [42]	2013	79	24	46	92	100	n.s.	7
Pearce et al. [26]	2012	62	11	37	30	n.s.	72	0
Vega et al. [40]	2008	69	8	43	27	100	n.s.	0
Alfredson et al. [25]	2011	73	16	47	18	74	74	3
Ruergård & Alfredson [38]	2014	79	47	52	12	n.s.	88	0
		72.4 ± 7.2	106	47.7 ± 5.5	34.0 ± 32.2.	91.3 ± 15.0	82.6 ± 8.7	2.0 ± 3.1

Year of publication (Year), Coleman Methodological Score (CMS), Number of patients (N), ^a^For this study N means number of tendons, Age (years), Followup period (FU) in months, Success rate (SR) in %, Patient satisfaction (PS) in %, and Complication rates (CR) in %. Values for FU, SR, and PS of the individual studies are presented as means. Summarized information of the specific operative techniques are presented in the last lines of the respective sections (CMS = unweighted mean. Age, FU, SR, PS, and CR = weighted means ± SD)

### Methodological quality

The mean CMS of all reviewed articles was 70.1 (range 53 to 84). We found no significant correlation between the CMS and the reported success rates (*r* = 0.04; *p* = 0.17). The correlation between the CMS and the year of publication was (*r* = 0.42, *p* = 0.07).

### Operative techniques

The mean success rate for all procedures was 83.4 % (range 66 to 100 %). Four studies didn’t report on the success rate (Table 1) [26, 29, 31, 38].The overall patient satisfaction was 77.5 % (range 60 to 95 %) and the complication rate was 6.3 % (range 0 to 23 %). In 2.4 % of the cases reoperation was necessary. In three cases (0.4 %) a total tendon rupture occurred during postoperative rehabilitation [4, 24, 25].

#### Open procedures

The results of open techniques were reported in 11 studies (604 tendons in 542 patients). The mean success rate was 78.9 % (range 66 to 100). The mean patient satisfaction rate was 78.1 % (range 66 to 95). The mean complication rate was 10.5 % (range 0 to 23) (Table 1) [4, 17, 19, 24, 29, 31, 32, 34, 35, 37, 41].

#### Minimally invasive techniques

Minimally invasive techniques were reported in nine studies (198 tendons in 172 patients). The mean success rate was 83.6 % (range 74 to 100). The mean patient satisfaction rate was 78.5 % (range 60 to 88). Complications occurred in 5.3 % of the cases (range 0 to 18; Table 2) [25, 26, 28, 30, 33, 36, 38–40].

#### Comparison of the techniques

Statistically, no difference was found between open and minimally invasive procedures regarding success rates (78.9 % and 83.6 %; *p* = 0.987) and patient satisfaction (78.1 % and 78.5 %; *p* = 0.211). Studies on open techniques reported a tendency to more complications than the studies on minimally invasive techniques (9.7 % and 3.1 %; *p* = 0.053) (Table 1).

The subgroup analyses for the success rates demonstrated superiority of minimally invasive paratenon debridement compared with percutaneous longitudinal tenotomy (*p* = 0.036). All other success rates comparisons were not statistically different (*p* = 0.083 to 0.916, Table 3). Higher patient satisfaction rates were detected for open paratenon debridement (94 %) compared with open intratendineous debridement (79.3 %, *p* < 0.003), gastrocnemius recession (61.6 %, *p* < 0.0001), percutaneous longitudinal tenotomy (81.1 %, *p* = 0.008), and minimally invasive paratenon debridement (82.5 %, *p* < 0.028). FHL tendon transfer/augmentation (91.3 %) was associated with a higher patient satisfaction rate than open intratendineous debridement (79.3 %, *p* = 0.006), gastrocnemius recession/lengthening (61.6 %, *p* = 0.001) and percutaneous longitudinal tenotomy (81.1 %, *p* = 0.017). The complication rates for FHL tendon transfer/augmentation (14.9 %) was higher than for open paratenon debridement (6 %, *p* = 0.048) and for minimally invasive paratenon debridement (2.0 %, *p* = 0.001; Table 2).

**Table 3:** Results of the statistical comparisons between the reviewed groups of different operative procedures

		SR	PS	CR
Open procedures vs.	Minimally invasive procedures	0.987	0.211	0.053
Open peritendineous debridement vs.	Open intratendineous debridement	0.250	**0.003** ^**a**^	0.062
	FHL transfer/augmentation	0.477	0.688	**0.048** ^**a**^
	Gastrocnemius recession	0.327	**0.0001** ^**a**^	0.557
	Percutaneous longitudinal tenotomy	0.083	**0.008** ^**a**^	0.846
	Minimally invasive paratenon debridement	0.866	**0.028** ^**a**^	0.188
Open intratendineous debridement vs.	FHL transfer/augmentation	0.692	**0.006** ^**b**^	0.913
	Gastrocnemius recession	0.882	0.264	0.145
	Percutaneous longitudinal tenotomy	0.752	0.756	0.121
	Minimally invasive paratenon debridement	0.130	0.507	0.160
FHL transfer/augmentation vs.	Gastrocnemius recession	0.620	**0.001** ^**a**^	0.144
	Percutaneous longitudinal tenotomy	0.311	**0.017** ^**a**^	0.097
	Minimally invasive paratenon debridement	0.266	0.064	**0.001** ^**b**^
Gastrocnemius recession vs.	Percutaneous longitudinal tenotomy	0.916	0.145	0.433
	Minimally invasive paratenon debridement	0.215	0.095	0.698
Percutaneous longitudinal tenotomy vs.	Minimally invasive paratenon debridement	**0.036** ^**b**^	0.814	0.077

Significant findings are presented in bold. The first line relates to Table 1. The rest of the table represents findings from Table 2. ^a^The significant value favours the technique which is described in the first column. ^b^The significant value favours the technique which is described in the second column

### Post-operative treatment

All studies but two [26, 33] reported on the postoperative treatment. The authors of open procedures allowed weight bearing after 2 weeks [19, 34], recommended the wearing of a cast for more than 6 weeks [4, 29, 32, 37, 41], and allowed competitive sports after two to six months [18, 24, 34, 35]. The authors of minimally invasive procedures recommended full weight bearing after one [36] to two weeks [19, 25, 31, 38–40], wearing of a cast for 6 weeks [28] and sports activity after 6 weeks [28] to three month [30].

## Discussion

This study systematically evaluates the literature for the effectiveness of operative treatment in patients with midportion Achilles tendinopathy. The analyses are based on success rates, patient satisfaction, and complication rates.

The most important finding of our study is that operative treatment is a successful option for midportion Achilles tendinopathy recalcitrant to non-operative treatment. Overall, this finding mirrors exactly the results of another recent systematic review (83.4 % vs. 83.5 %) [21]. That review, however, is biased by including also retrocalcaneal bursitis and case reports. A systematic review for retrocalcaneal bursitis surgery presented success rates of 91 % for endoscopic and 73 % for open procedures [43]. Our results are superior to a critical Achilles tendinopathy review from 2001 (mean success rate = 77 %) [20].

We specifically addressed midportion Achilles tendinopathy. Depending from the used technique, success rates vary between 73 and 100 %. Even if the reviewed literature did not definitely describe the pathologic stage of the treated tendons, one could argue that more advanced stages of Achilles tendon degeneration will lead the treating surgeon to indicate more complex operative treatments. In practice a surgeon would not randomly choose between minimally invasive tendon debridement and FHL augmentation but would choose the latter procedure for a more severe case. Consequently, different stages of Achilles tendon lesions may differ in their response to a specific operative technique; e.g. advanced stages of Achilles tendon degeneration with impaired tendon quality may in principle be better treated with an augmentation, while an isolated minimally invasive paratenon debridement may not be sufficient for that advanced pathology. Most minimally invasive techniques focus mainly the peritendineous tissues and intend to eliminate neovascularization with its accompanying nerves as a cause of pain and disease progression [10]. So, minimally invasive approaches in principle address pain, while open techniques aim to treat the degenerated tendon tissue. For the results of the analyses of the different operative techniques (subgroup analyses), a respective selection bias has to be stated. Considering these limitation this systematic review shows that there is no statistical difference between open procedures and the minimally invasive techniques regarding success rates (78.9 % vs. 83.6 %; *p* = 0.987) and patient satisfaction (78.1 % vs. 78.5 %; *p* = 0.211), but the complication rate is tendentially higher following open surgery (10.5 % vs. 5.3 %; *p* = 0.053).

The subgroup analysis showed superiority of the minimally invasive paratenon debridement over percutaneous longitudinal tenotomy (91.3 % vs. 81.1 %; *p* = 0.036). The higher patient satisfaction and lower complication rates following paratenon debridement (Table 3) are based on only one report and therefore needs further substantiation in future research. Lowest success rates are presented for gastrocnemius recession (61.6 %; Table 3). Comparison of the complication rates favored minimally invasive paratenon debridement (2.0 %; Tables 2 and 3).

According to previous reports we decided to use the CMS to make outcomes comparable [25, 44]. A previous critical review on operative midportion Achilles tendinopathy treatment reported on a mean CMS of 37.6 (range 2 to 74) [20] while another systematic review included both, Achilles tendinopathy and retrocalcaneal bursitis and found a mean CMS of 40.1 (range 2 to 79). As a result of our more strict inclusion criteria the studies included in the present investigation reached a mean score of 70.5 (range 51 to 85). Previously, the methodological quality of the midportion Achilles tendinopathy research has been shown to directly influence the reported outcome [20, 21] and an increase in the CMS was connected with a decrease in the reported success rate. In our systematic review, however, CMS and success rate did not correlate significantly (*r* = 0.04; *p* = 0.17).

A positive linear correlation between the CMS and the year of publication was also previously stated [20, 21]. This means that the quality of the published articles were thought to increase over the time. Our analyses found a trend in the same direction (*r* = 0.42, *p* = 0.07). One reason for missing the significance level may be that our data were based on material mainly from the past 12 years, representing a time period of generally higher methodological quality research. Additionally, case reports and small case series were excluded in our review.

Three studies reported different results for subpopulations and reported higher success and lower complication rates for male patients and also for athletic patients [25, 34, 35].

Strengths of this systematic review are the strict inclusion criteria and the fact that all the included studies were prospective.

Randomized comparisons between open and minimally invasive procedures referring to the same severity/grade of Achilles tendinopathy are still missing and should be performed in the future and meta-analyses are required to definitely demonstrate the worth of the different techniques [45]. Even if we tried to detect all relevant articles in our search algorithm, studies may have been excluded based on their choice of terminology. A similar criticism relates to the post-operative treatment. The short followup period of several studies is another concern. In studies on conservative treatment followup periods with more than five years [44] are available, while the shortest followup period in our included studies was six month [19]. For the classification of results authors often used questionnaires which were not region specific or validated [46–49] and this detail could also bias the results and has to be adequately addressed in future research. In the future researchers should use valid, reliable, and sensitive outcome measures like the VISA-A questionnaire [47] to longitudinally and objectively quantify the effects of their interventions. We demand randomized controlled studies focusing on the sole effect of operative treatments. This would allow being more conclusive regarding the best applicable treatment for a specific patient suffering from recalcitrant midportion Achilles tendinopathy.

## Conclusion

Operative treatments seem to be a good option for midportion Achilles tendinopathy patients, when conservative treatment fails.

Resulting from the lower complication rate, this systematic review recommends minimally invasive surgery as the primary operative treatment option. However, a stage adapted procedure is recommended.

### Ethics and consent to participate

Not applicable as this is a systematic review of published studies.

### Consent to publish

Not applicable.

### Availability of data and materials

All the data supporting our findings is contained within the manuscript.

## Abbreviations

CMS: Coleman methodology scale
ESWT: Extracorporeal Shock Wave Therapy
FHL: Flexor hallucis longus
VISA-A: Victorian institute of sports assessment–Achilles tendon
Vs.: versus
